# Efficacy and safety of thoracic radiotherapy combined with anti-angiogenic therapy and immunochemotherapy for advanced non-small cell lung cancer patients: a retrospective study

**DOI:** 10.3389/fonc.2025.1640306

**Published:** 2025-09-23

**Authors:** Kai Gai, Xuexin Shi, Fei Xu, Xia Ding, Lu Liu, Longjie Zhang, Hao Wang, Ling Chen

**Affiliations:** ^1^ Department of Oncology, Qingdao Municipal Hospital, Qingdao, Shandong, China; ^2^ Department of Thoracic Surgery, Qingdao Municipal Hospital, Qingdao, Shandong, China

**Keywords:** lung cancer, NSCLC, radiotherapy, anti-angiogenic therapy, immunotherapy, immune checkpoint inhibitor, combination therapy

## Abstract

**Purpose:**

To assess the efficacy and safety of radiotherapy combined with anti-angiogenic therapy, immune checkpoint inhibitors, and chemotherapy for advanced non-small cell lung cancer (NSCLC).

**Methods:**

Patients who have received at least two cycles of quadruple therapy were Progression-free survival (PFS), overall survival (OS), and treatment-related side effects were comprehensively evaluated using R language and the chi-square test.

**Results:**

Seventy-four patients were enrolled and divided into two groups (A and B) based on whether they had received radiotherapy. The incidence rate of adverse events—such as radiation-related pneumonitis, pneumonia, thrombocytopenia, cough, panting, fatigue, and radiodermatitis—were significantly higher in patients receiving radiotherapy. A survival analysis comparing the experimental and control groups revealed that the addition of radiotherapy played a positive role in extending PFS and OS, with statistically significant results observed for OS (HR(95%CI)=0.51[0.283, 0.919]; *p* =0.019). The risk of radiation-related pneumonitis was significantly higher than in the control group (*p <*0.001) and was associated with a negative impact on prognosis; a similar trend was also observed for pneumonia (*p*=0.041) and thrombocytopenia (*p <*0.001).

**Conclusions:**

Sequential radiotherapy after quadruple therapy can prolong survival in patients with advanced NSCLC. However, special attention should be paid to treatment-related side effects such as pneumonitis, pneumonia, and thrombocytopenia, which may negatively affect prognosis.

## Introduction

According to the latest statistics, lung cancer has the highest morbidity and mortality among all malignant tumors in China, with non-small cell lung cancer (NSCLC) accounting for approximately 85% of all lung cancers (1). The first-line treatment for NSCLC has been substantially revolutionized since immunotherapy became a cornerstone. Currently, immune checkpoint inhibitors (ICIs) combined with platinum-doublet chemotherapy have become popular treatment options for advanced NSCLC lacking driver gene mutations (2).

Before immunotherapy became the main therapeutic modality in oncology, radiotherapy and anti-angiogenic therapy played irreplaceable roles in the treatment of advanced NSCLC (3). However, the role of radiotherapy remains controversial. Only limited evidence suggests that radiotherapy and anti-angiogenic therapy may improve the outcomes of immunotherapy for advanced NSCLC. For instance, a secondary analysis of the KEYNOTE-001 phase 1 trial showed that patients with NSCLC who received radiotherapy before pembrolizumab had longer progression-free survival (PFS) and overall survival (OS) than those who did not receive prior radiotherapy, with an acceptable safety profile (4).

In addition, the IMpower150 trial explored the value of bevacizumab in combination with atezolizumab and chemotherapy as a first-line treatment for advanced non-squamous NSCLC, and patients who received combination therapy demonstrated a survival advantage (5). Although the PACIFIC study established 12 months of consolidation therapy with durvalumab after concurrent chemoradiotherapy (cCRT) as the standard treatment for patients with unresectable stage III NSCLC worldwide (6), there remains a lack of clinical research on the combined use of radiotherapy, anti-angiogenic therapy, and immunochemotherapy for advanced NSCLC. This study was designed to explore the significance of incorporating radiotherapy into combination therapy.

## Methods

### Patients screening

Patients with advanced NSCLC confirmed by pathology at Qingdao Municipal Hospital were enrolled between January 2020 and January 2023. PD-1 inhibitors, thoracic radiotherapy, anti-angiogenic inhibitors, and chemotherapy were prescribed as first-line treatment. The main difference between the experimental group (Group A) and the control group (Group B) was whether or not thoracic radiotherapy was administered.

The selection criteria for patients were as follows: (1) aged between 18 and 80 years; (2) TNM stage IV or advanced NSCLC; (3) Eastern Cooperative Oncology Group (ECOG) score ≤1; (4) prescribed PD-1 inhibitors, thoracic radiotherapy, and anti-angiogenic treatments; (5) no other malignancies observed; (6) no significant cardiovascular abnormalities; (7) no obvious organ dysfunction; (8) complete clinical information; and (9) at least two cycles of quadruple therapy administered. The exclusion criteria were listed as follows: (1) mutations in EGFR or related genes; (2) prior administration of ICI-based immunotherapy; and (3) disease progression considered ineligible.

### Treatment regimens

Anti-angiogenic therapy and PD-1 inhibitors combined with platinum-doublet chemotherapy were used as first-line treatment options for patients with advanced NSCLC. Patients with adenocarcinoma received bevacizumab, whereas those with squamous cell carcinoma received endostar. Immunotherapy was limited to PD-1 inhibitors, with no additional restrictions.

Thoracic radiotherapy was administered after at least two cycles of quadruple therapy or when the patient’s efficacy evaluation indicated stable disease (SD) and a trend of tumor enlargement was observed. The patient received a radiotherapy prescription dose of at least 30 Gy/10 f (a total dose of 30 gray delivered in 10 fractions), and subsequent decisions on whether to administer a local boost were based on tumor regression and treatment toxicities, with the total dose not exceeding 45 Gy/15 f or 60 Gy/30 f. Gross tumor volume (GTV) was determined through CT scanning, and delineation of the target area was completed on the CT pulmonary window. Furthermore, the planning target volume (PTV) was obtained by expanding the GTV by 1.0 cm. Based on CT simulation and 3D planning systems, all patients received intensity-modulated radiotherapy (IMRT).

Organ protection and dose restrictions were as follows (7–14): whole lung: V20 ≤30%, V30 ≤20%, V5 ≤60%, mean lung dose (MLD) <13 Gy; V20 was considered the primary dose-limiting factor in radiotherapeutic parameters; spinal cord: Dmax ≤44 Gy; heart: V40 ≤35%, D100 ≤30 Gy, and D50 <40 Gy. The risk of radiation-related pneumonitis should be considered a treatment-related side effect requiring prioritization (8, 9, 11–14).

The chemotherapy regimen should not include drugs such as gemcitabine, which increase the risk of radiation pneumonitis, whereas other agents are acceptable. Chemotherapy was temporarily postponed during radiotherapy. Unless grade 3 or higher treatment-related adverse reactions occurred, treatment was not interrupted. In such cases, the dosage of chemotherapy drugs was reduced by 25%. If the side effects did not improve, chemotherapy was discontinued. Hormonal, expectorant, and antiasthmatic drugs were administered as supportive treatments throughout the radiotherapy process.

### Efficacy and safety evaluation

Based on CT images, immune-Modified Response Evaluation Criteria In Solid Tumors (imRECIST) were used to assess treatment response (15), whereas adverse events were evaluated at least once every cycle according to the Common Terminology Criteria for Adverse Events (CTCAE) (16, 17).

### Follow-up and survival assessment

Follow-up was conducted until November 2023 or until patient death. Follow-up examinations included assessments of disease progression status, physical examination, blood counts, biochemical profiles, and CT images. Similar to previous studies (5, 18–20), overall survival (OS) was considered the primary endpoint and observation indicator, while progression-free survival (PFS) and treatment tolerance were listed as secondary endpoints. OS and PFS were assessed according to follow-up records. OS was defined as the time interval from initiation of first-line treatment to death or last follow-up, with no restrictions on the cause of death. PFS was defined as the time interval from initiation of first-line treatment to disease progression or death due to NSCLC, excluding other causes.

### Statistical analysis

Differences between the experimental and control groups were determined using Pearson’s chi-square or Wilcoxon tests (7). OS and PFS were analyzed using the Kaplan–Meier method, and univariate analysis was performed using R software (Version 4.4.2). All reported p-values were two-sided, and a p-value less than 0.05 was considered statistically significant. All statistical analyses were performed using SPSS software (Version 27.0).

## Results

### Baselines of all enrolled patients before radiotherapy

Seventy-four patients were enrolled and divided into two groups, A and B, based on whether they had received radiotherapy after two cycles of quadruple anti-tumor therapy (**Table 1**). All patients were EGFR mutation-negative. Among all patients, the proportion of males was higher; the proportion of males in Group A was 69.8%, while in Group B it was 71%. Upon reviewing the baseline characteristics of both groups, no statistically significant differences were observed, except for the proportion of patients with stable disease (SD) status (**Table 1**; *p* = 0.017). Detailed clinical information for each patient, including performance status, smoking history, comorbidities, and treatments, is included in **Supplementary Table S1**. Subgroup analysis was first performed. The PFS and OS of patients with different histological subtypes (**Supplementary Figures S1A, D**), PD-L1 expression levels (**Supplementary Figures S1B, E**), and smoking status (**Supplementary Figures S1C, F**) are shown in **Supplementary Figure S1**.

**Table 1:** Baseline characteristics of all enrolled patients before radiotherapy.

Variable	No. (%)		
	Group A (n = 43)	Group B (n = 31)	P value
Age(y), median	65	61	0.896
<60 y	16 (37.2)	12 (38.7)	0.930
≥60 y	27 (62.8)	19 (61.3)	0.949
Sex			0.911
Male	30 (69.8)	22 (71.0)	0.963
Female	13 (31.2)	9 (29.0)	0.935
Alcohol smoker			0.557
Yes	22 (51.2)	18 (58.1)	0.749
No	21 (48.8)	13 (41.9)	0.719
Histological subtype			0.268
Adenocarcinoma	21 (48.8)	14 (45.2)	0.851
Squamous carcinoma	19 (44.2)	17 (54.8)	0.597
Others	3 (7.0)	0 (0)	0.395
Tumor stage			0.973
T1	2 (4.7)	1 (3.2)	1.000
T2	25 (58.1)	18 (58.1)	0.997
T3	14 (32.6)	11 (35.5)	0.854
T4	2 (4.7)	1 (3.2)	1.000
Lymph node status			0.003
N0	5 (11.6)	0 (0)	0.166
N1	14 (32.6)	5 (16.1)	0.214
N2	20 (46.5)	13 (41.9)	0.808
N3	4 (9.3)	13 (41.9)	0.010
Metastasis status			0.977
M1a	7 (16.3)	5 (16.1)	0.988
M1b	12 (27.9)	8 (25.8)	0.879
M1c	24 (55.8)	18 (58.1)	0.919
Distant metastasis			0.981
Brain	11 (25.6)	7 (22.5)	0.816
Lung	27 (62.8)	20 (64.5)	0.943
Bone	13 (30.2)	11 (35.5)	0.735
Liver	6 (14.0)	5 (16.1)	1.000
Adrenal	7 (16.3)	4 (12.9)	0.985
Clinical stage			0.452
IVA	19 (44.2)	11 (35.5)	0.623
IVB	24 (55.8)	20 (64.5)	0.706
Efficacy evaluation			0.001
SD	27 (62.8)	6 (19.4)	0.017
PR	16 (37.2)	23 (74.2)	0.084
PD	0	2 (6.5)	0.185
PD-L1 expression			0.330
TPS 1-49%	32 (74.4)	26 (83.9)	0.735
TPS ≥50%	11 (25.6)	5 (16.1)	0.431

All the information mentioned above was collected before the patient underwent radiotherapy.

### Assessment of efficacy and adverse events

All 43 patients in Group A received 10 sessions of radiotherapy, with a total dose of 30 Gy. After undergoing radiotherapy, 23 patients in Group A with stable disease (SD) reached partial response (PR), and 4 patients remained in SD status. The primary tumors of 16 patients with PR had further shrunk compared to baseline.

**Figure 1:**
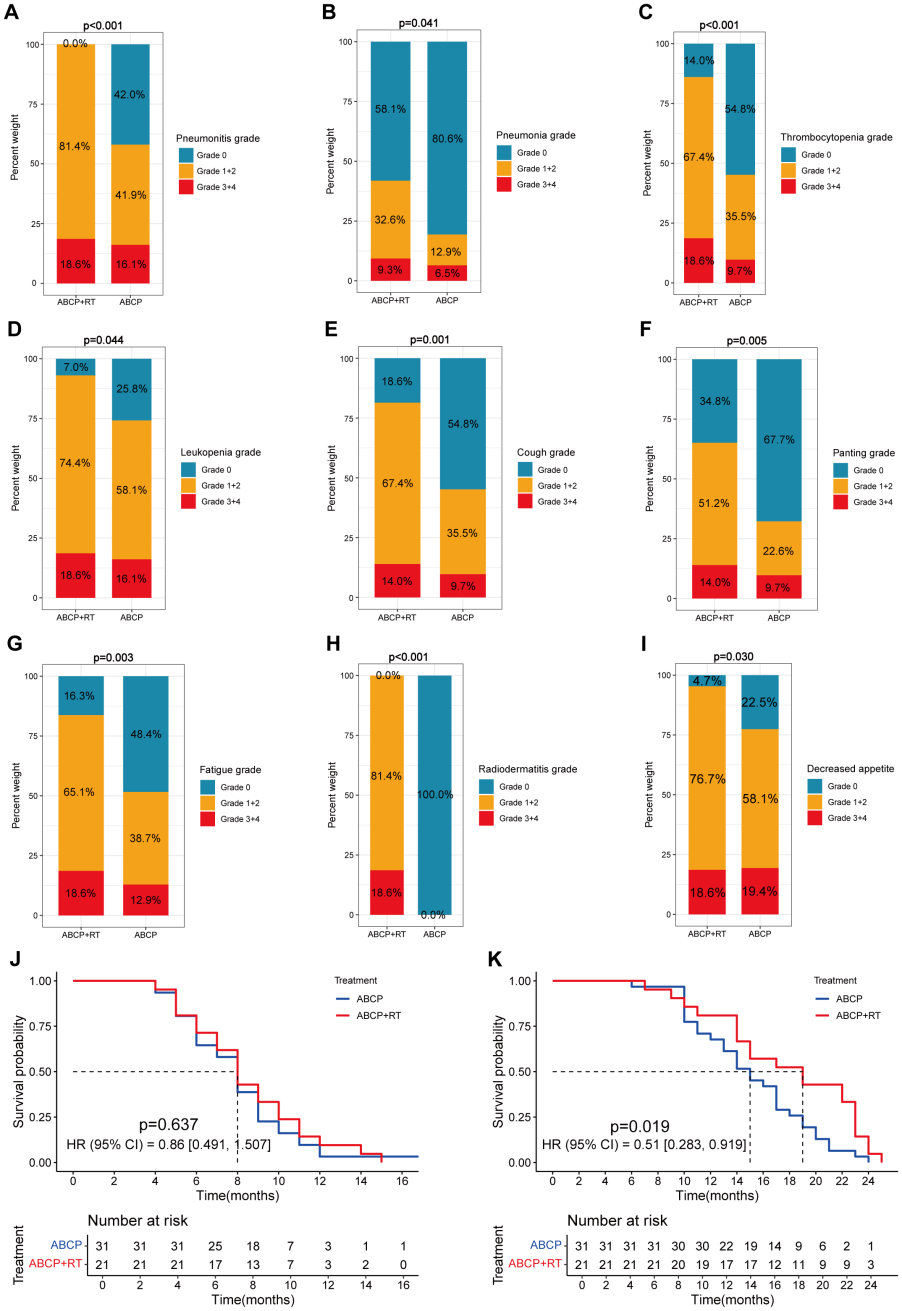
Differences in clinical features between lung cancer patients treated with ABCP in combination with RT and those treated with ABCP alone. **(A–I)** Percentage plots showing the proportion of patients with grade 0, grade 1 + 2 and grade 3 + 4 of pneumonitis **(A)**, pneumonia **(B)**, thrombocytopenia **(C)**, leukopenia **(D)**, cough **(E)**, panting **(F)**, fatigue **(G)**, radiodermatitis **(H)**, and decreased appetite **(I)** in the ABCP+RT and ABCP-only treatment groups. Blue, orange, and red bars represent grades 0, 1–2, and 3–4, respectively. The x-axes indicate treatment groups, while the y-axes show the percentage of each grade. Notes for each figure are shown on the right side of the corresponding panel. **(J)** PFS curves of patients treated with ABCP alone versus ABCP combined with RT. **(K)** OS curves of patients treated with ABCP alone versus ABCP combined with RT. Blue lines represent patients treated with ABCP alone, and red lines represent those treated with ABCP+RT. The x-axes indicate survival time; the y-axes indicate survival probability. Grouping status is indicated at the bottom of each chart. p < 0.05 in the log-rank test was considered statistically significant. PFS, progression-free survival; OS, overall survival; RT, radiotherapy; ABCP, atezolizumab, bevacizumab, carboplatin, and paclitaxel.

Following the addition of radiotherapy to the quadruple therapy regimen, the risk of all toxicities was higher than in the control group (**Table 2**, **Figures 1A–I**). All p-values from the chi-square tests comparing the two groups are listed on the right side of the **Table 2**. Among all patients, hematological toxicities remained the most common treatment-related side effects (**Table 2**). For all hematological toxicities, the risk of thrombocytopenia in the experimental group (Group A) was significantly higher than in the control group (Group B) (**Figure 1C**; 86.0% vs. 45.2%; *p* < 0.001). Similar increases in risk were observed for pneumonitis (**Figure 1A**; 100% vs. 58.06%; *p* < 0.001), cough (**Figure 1E**; 81.4% vs. 45.2%; *p* = 0.001), panting (**Figure 1F**; 65.2% vs. 32.3%; *p* = 0.005), and decreased appetite (**Figure 1I**; 95.3% vs. 77.5%; *p* = 0.030) (**Table 2**).

**Table 2:** Information on all treatment-related adverse events.

Events	Grade 1 + 2			Grade 3 + 4			Grade 1-4		
	ABCP + RT (n = 43)	ABCP (n = 31)	P-value	ABCP + RT (n = 43)	ABCP (n = 31)	P-value	ABCP + RT (n = 43)	ABCP (n = 31)	P-value
Hypoalbuminemia	33 (76.7)	21 (67.7)	0.390	8 (18.6)	6 (19.4)	0.935	41 (95.3)	27 (87.1)	0.230
Anemia	32 (74.4)	20 (64.5)	0.358	7 (16.3)	6 (19.4)	0.732	39 (90.7)	26 (83.9)	0.478
Leukopenia	32 (74.4)	18 (58.1)	0.138	8 (18.6)	5 (16.1)	0.782	40 (93.0)	23 (74.2)	0.044
Lymphocytopenia	30 (69.8)	19 (61.3)	0.447	7 (16.3)	4 (12.9)	0.752	37 (86.1)	23 (74.2)	0.199
Neutropenia	27 (62.8)	17 (54.8)	0.492	7 (16.3)	5 (16.1)	0.986	34 (79.1)	22 (70.9)	0.423
Thrombocytopenia	29 (67.4)	11 (35.5)	0.006	8 (18.6)	3 (9.7)	0.340	37 (86)	14 (45.2)	<0.001
Pancytopenia	21 (48.8)	9 (29.0)	0.087	5 (11.6)	3 (9.7)	1.000	26 (60.4)	12 (38.7)	0.065
Pneumonitis	35 (81.4)	13 (41.9)	<0.001	8 (18.6)	5 (16.1)	0.782	43 (100.0)	18 (58.0)	<0.001
Pneumonia	14 (32.6)	4 (12.9)	0.060	4 (9.3)	2 (6.5)	0.627	18 (41.9)	6 (19.4)	0.041
Respiratory tract infection	16 (37.2)	6 (19.4)	0.097	4 (9.3)	3 (9.7)	1.000	20 (46.5)	9 (29.1)	0.129
Pulmonary embolism	1 (2.3)	0 (0)	1.000	2 (4.7)	1 (3.2)	1.000	3 (7.0)	1 (3.2)	0.635
Cough	29 (67.4)	11 (35.5)	0.006	6 (14.0)	3 (9.7)	0.580	35 (81.4)	14 (45.2)	0.001
Panting	22 (51.2)	7 (22.6)	0.013	6 (14.0)	3 (9.7)	0.726	28 (65.2)	10 (32.3)	0.005
Hemoptysis	4 (9.3)	1 (3.2)	0.392	2 (4.7)	1 (3.2)	1.000	6 (14.0)	2 (6.5)	0.455
Esophagitis	13 (30.2)	7 (22.6)	0.465	4 (9.3)	2 (6.5)	0.627	17 (39.5)	9 (29.1)	0.350
Stomatitis	12 (27.9)	6 (19.4)	0.398	2 (4.7)	1 (3.2)	1.000	14 (32.6)	7 (22.6)	0.348
Colitis	8 (18.6)	5 (16.1)	0.782	2 (4.7)	2 (6.5)	1.000	10 (23.3)	7 (22.6)	0.946
Hepatitis	2 (4.7)	0 (0)	0.506	1 (2.3)	1 (3.2)	1.000	3 (7.0)	1 (3.2)	0.635
Pancreatitis	1 (2.3)	1 (3.2)	1.000	1 (2.3)	0 (0)	1.000	2 (4.7)	1 (3.2)	1.000
Decreased appetite	33 (76.7)	18 (58.1)	0.087	8 (18.6)	6 (19.4)	0.935	41 (95.3)	24 (77.5)	0.030
Nausea	31 (72.1)	18 (58.1)	0.208	7 (16.3)	5 (16.1)	0.986	38 (88.4)	23 (74.2)	0.114
Vomiting	25 (58.1)	12 (38.7)	0.099	5 (11.6)	4 (12.9)	1.000	30 (69.7)	16 (51.6)	0.112
Constipation	23 (53.4)	11 (35.5)	0.125	6 (14.0)	3 (9.7)	0.726	29 (67.4)	14 (45.2)	0.055
Diarrhea	16 (37.2)	9 (29.0)	0.463	5 (11.6)	3 (9.7)	1.000	21 (48.8)	12 (38.7)	0.387
Proteinuria	9 (20.9)	5 (16.1)	0.603	3 (7.0)	2 (6.5)	1.000	12 (27.9)	7 (22.6)	0.605
Diabetes mellitus	4 (9.3)	2 (6.5)	1.000	1 (2.3)	0 (0)	1.000	5 (11.6)	2 (6.5)	0.692
Hypertension	16 (37.2)	10 (32.3)	0.660	4 (9.3)	3 (9.7)	1.000	20 (46.5)	13 (42.0)	0.696
Cerebrovascular accident	2 (4.7)	1 (3.2)	1.000	1 (2.3)	0 (0)	1.000	3 (7.0)	1 (3.2)	0.635
Paresthesia	7 (16.3)	3 (9.7)	0.505	2 (4.7)	2 (6.5)	1.000	9 (20.9)	5 (16.1)	0.603
Ataxia	1 (2.3)	0 (0)	1.000	1 (2.3)	1 (3.2)	1.000	2 (4.7)	1 (3.2)	1.000
Cerebral ischemia	2 (4.7)	1 (3.2)	1.000	1 (2.3)	0 (0)	1.000	3 (7.0)	1 (3.2)	0.635
Depressed level of consciousness	4 (9.3)	1 (3.2)	0.386	3 (7.0)	2 (6.5)	1.000	7 (16.3)	3 (9.7)	0.505
Dizziness	5 (11.6)	2 (6.5)	0.692	4 (9.3)	3 (9.7)	1.000	9 (20.9)	5 (16.1)	0.603
Headache	7 (16.3)	4 (12.9)	0.752	3 (7.0)	2 (6.5)	1.000	10 (23.3)	6 (19.4)	0.688
Peripheral neuropathy	6 (14.0)	4 (12.9)	1.000	2 (4.7)	1 (3.2)	1.000	8 (18.6)	5 (16.1)	0.782
Arthralgia	5 (11.6)	3 (9.7)	1.000	2 (4.7)	0 (0)	0.506	7 (16.3)	3 (9.7)	0.505
Myalgia	4 (13.8)	3 (9.7)	1.000	1 (3.4)	1 (3.4)	1.000	5 (11.6)	4 (12.9)	1.000
ALT increased	6 (14.0)	4 (12.9)	1.000	2 (4.7)	1 (3.2)	1.000	8 (18.6)	5 (16.1)	0.782
AST increased	5 (11.6)	3 (9.7)	1.000	2 (4.7)	1 (3.2)	1.000	7 (16.3)	4 (12.9)	0.752
Transaminases increased	2 (4.7)	0 (0)	0.506	1 (2.3)	1 (3.2)	1.000	3 (7.0)	1 (3.2)	0.635
Hyperkalemia	3 (10.3)	2 (6.5)	1.000	2 (6.9)	1 (3.2)	1.000	5 (11.6)	3 (9.7)	1.000
Hypokalemia	2 (4.7)	1 (3.2)	1.000	1 (2.3)	1 (3.2)	1.000	3 (7.0)	2 (6.5)	1.000
Hyponatremia	1 (2.3)	1 (3.2)	1.000	1 (2.3)	0 (0)	1.000	2 (4.7)	1 (3.2)	1.000
Alopecia	30 (69.8)	21 (67.7)	1.000	8 (18.6)	6 (19.4)	0.935	38 (88.4)	27 (87.1)	1.000
Fatigue	28 (65.1)	12 (38.7)	0.025	8 (18.6)	4 (12.9)	0.750	36 (83.7)	16 (51.6)	0.003
Epistaxis	10 (23.3)	6 (19.4)	0.688	2 (4.7)	1 (3.2)	1.000	12 (28.0)	7 (22.6)	0.605
Decreased weight	5 (11.6)	3 (9.7)	1.000	3 (7.0)	2 (6.9)	1.000	8 (18.6)	5 (16.1)	0.782
Dehydration	7 (16.3)	3 (9.7)	0.505	2 (4.7)	0 (0)	0.506	9 (20.9)	3 (9.7)	0.338
Radiodermatitis	35 (81.4)	0 (0)	<0.001	8 (18.6)	0 (0)	<0.001	43 (100.0)	0 (0.0)	<0.001
Rash	14 (32.6)	7 (22.6)	0.348	5 (11.6)	3 (9.7)	1.000	19 (44.2)	10 (32.3)	0.300
Pyrexia	15 (34.9)	8 (25.8)	0.405	7 (16.3)	5 (16.1)	0.986	22 (51.2)	13 (41.9)	0.433
Hypothyroidism	9 (20.9)	6 (19.4)	0.868	3 (7.0)	2 (6.5)	1.000	12 (27.9)	8 (25.8)	0.841
Hyperthyroidism	6 (14.0)	3 (10.3)	0.726	2 (4.7)	1 (3.4)	1.000	8 (18.6)	4 (12.9)	0.750

Among patients receiving radiotherapy, the risk of radiation-specific side effects, such as fatigue (**Figure 1G**; *p* = 0.003) and radiodermatitis (**Figure 1H**; *p* < 0.001), was also significantly higher than in the control group.

### Results of survival analysis

**Figure 2:**
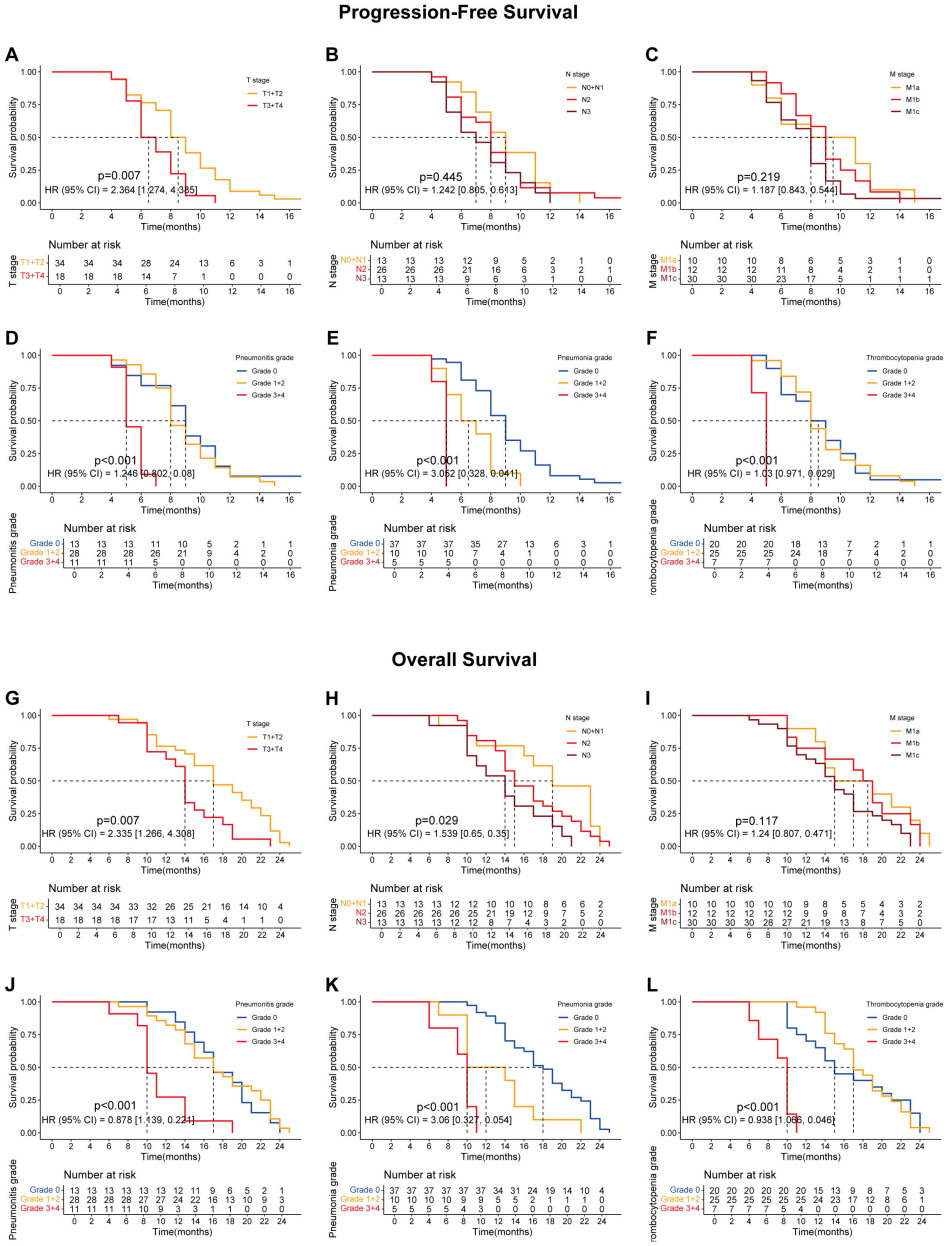
Survival curves showing progression-free survival (PFS) and overall survival (OS) for all lung cancer patients included in this study, including those treated with the combination of ABCP and radiotherapy (RT) and those treated with ABCP alone. **(A)** PFS curves of patients with tumors at different T stages. The orange and red lines represent patients with stage T1–T2 and T3–T4 tumors, respectively. **(B)** PFS curves of patients with tumors at different N stages. The orange, red, and dark red lines represent patients with stage N0–N1, N2, and N3 tumors, respectively. **(C)** PFS curves of patients with tumors at different M stages. The orange, red, and dark red lines represent patients with stage M1a, M1b, and M1c tumors, respectively. **(D)** PFS curves of patients with different grades of pneumonitis. The blue, orange, and red lines represent patients with grade 0, grade 1–2, and grade 3–4 pneumonitis, respectively. **(E)** PFS curves of patients with different grades of pneumonia. The blue, orange, and red lines represent patients with grade 0, grade 1–2, and grade 3–4 pneumonia, respectively. **(F)** PFS curves of patients with different grades of thrombocytopenia. The blue, orange, and red lines represent patients with grade 0, grade 1–2, and grade 3–4 thrombocytopenia, respectively. **(G)** OS curves of patients with tumors at different T stages. The orange and red lines represent patients with stage T1–T2 and T3–T4 tumors, respectively. **(H)** OS curves of patients with tumors at different N stages. The orange, red, and dark red lines represent patients with stage N0–N1, N2, and N3 tumors, respectively. **(I)** OS curves of patients with tumors at different M stages. The orange, red, and dark red lines represent patients with stage M1a, M1b, and M1c tumors, respectively. **(J)** OS curves of patients with different grades of pneumonitis. The blue, orange, and red lines represent patients with grade 0, grade 1–2, and grade 3–4 pneumonitis, respectively. **(K)** OS curves of patients with different grades of pneumonia. The blue, orange, and red lines represent patients with grade 0, grade 1–2, and grade 3–4 pneumonia, respectively. **(L)** OS curves of patients with different grades of thrombocytopenia. The blue, orange, and red lines represent patients with grade 0, grade 1–2, and grade 3–4 thrombocytopenia, respectively. The x-axes indicate survival time, and the y-axes indicate survival probability. Grouping status is shown at the bottom of each chart. p < 0.05 in the log-rank test was considered statistically significant. PFS, progression-free survival; OS, overall survival; ABCP, atezolizumab, bevacizumab, carboplatin, and paclitaxel.

**Figure 3:**
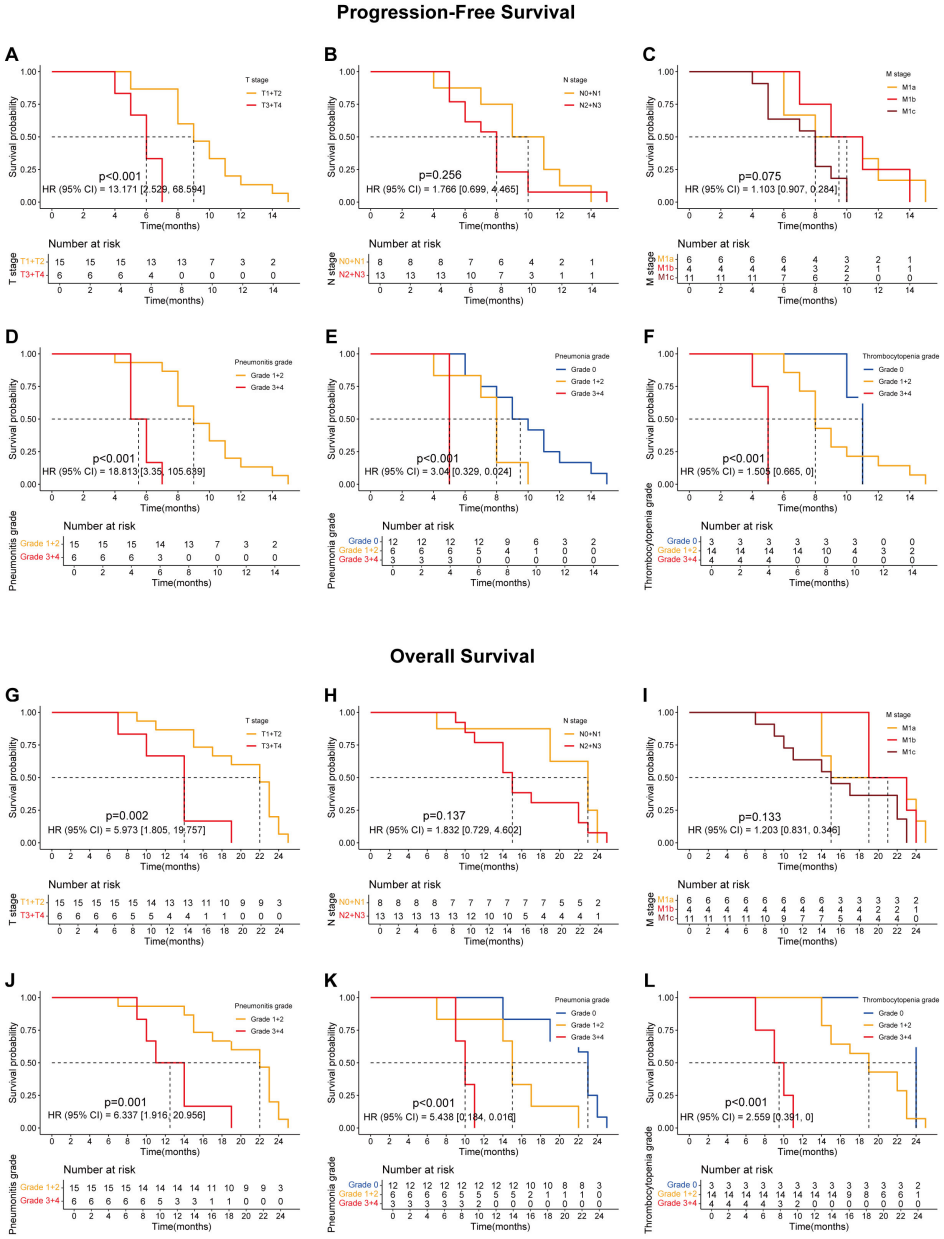
Survival curves showing progression-free survival (PFS) and overall survival (OS) of lung cancer patients treated with the combination of ABCP and radiotherapy (RT). **(A)** PFS curves of patients with tumors at different T stages. The orange and red lines represent patients with stage T1–T2 and T3–T4 tumors, respectively. **(B)** PFS curves of patients with tumors at different N stages. The orange and red lines represent patients with stage N0–N1 and N2–N3 tumors, respectively. **(C)** PFS curves of patients with tumors at different M stages. The orange, red, and dark red lines represent patients with stage M1a, M1b, and M1c tumors, respectively. **(D)** PFS curves of patients with different grades of pneumonitis. The orange and red lines represent patients with grade 1–2 and grade 3–4 pneumonitis, respectively. **(E)** PFS curves of patients with different grades of pneumonia. The blue, orange, and red lines represent patients with grade 0, grade 1–2, and grade 3–4 pneumonia, respectively. **(F)** PFS curves of patients with different grades of thrombocytopenia. The blue, orange, and red lines represent patients with grade 0, grade 1–2, and grade 3–4 thrombocytopenia, respectively. **(G)** OS curves of patients with tumors at different T stages. The orange and red lines represent patients with stage T1–T2 and T3–T4 tumors, respectively. **(H)** OS curves of patients with tumors at different N stages. The orange and red lines represent patients with stage N0–N1 and N2–N3 tumors, respectively. **(I)** OS curves of patients with tumors at different M stages. The orange, red, and dark red lines represent patients with stage M1a, M1b, and M1c tumors, respectively. **(J)** OS curves of patients with different grades of pneumonitis. The orange and red lines represent patients with grade 1–2 and grade 3–4 pneumonitis, respectively. **(K)** OS curves of patients with different grades of pneumonia. The blue, orange, and red lines represent patients with grade 0, grade 1–2, and grade 3–4 pneumonia, respectively. **(L)** OS curves of patients with different grades of thrombocytopenia. The blue, orange, and red lines represent patients with grade 0, grade 1–2, and grade 3–4 thrombocytopenia, respectively. The x-axes indicate survival time, and the y-axes indicate survival probability. Grouping status is shown at the bottom of each chart. p < 0.05 in the log-rank test was considered statistically significant. PFS, progression-free survival; OS, overall survival; ABCP, atezolizumab, bevacizumab, carboplatin, and paclitaxel.

**Figure 4:**
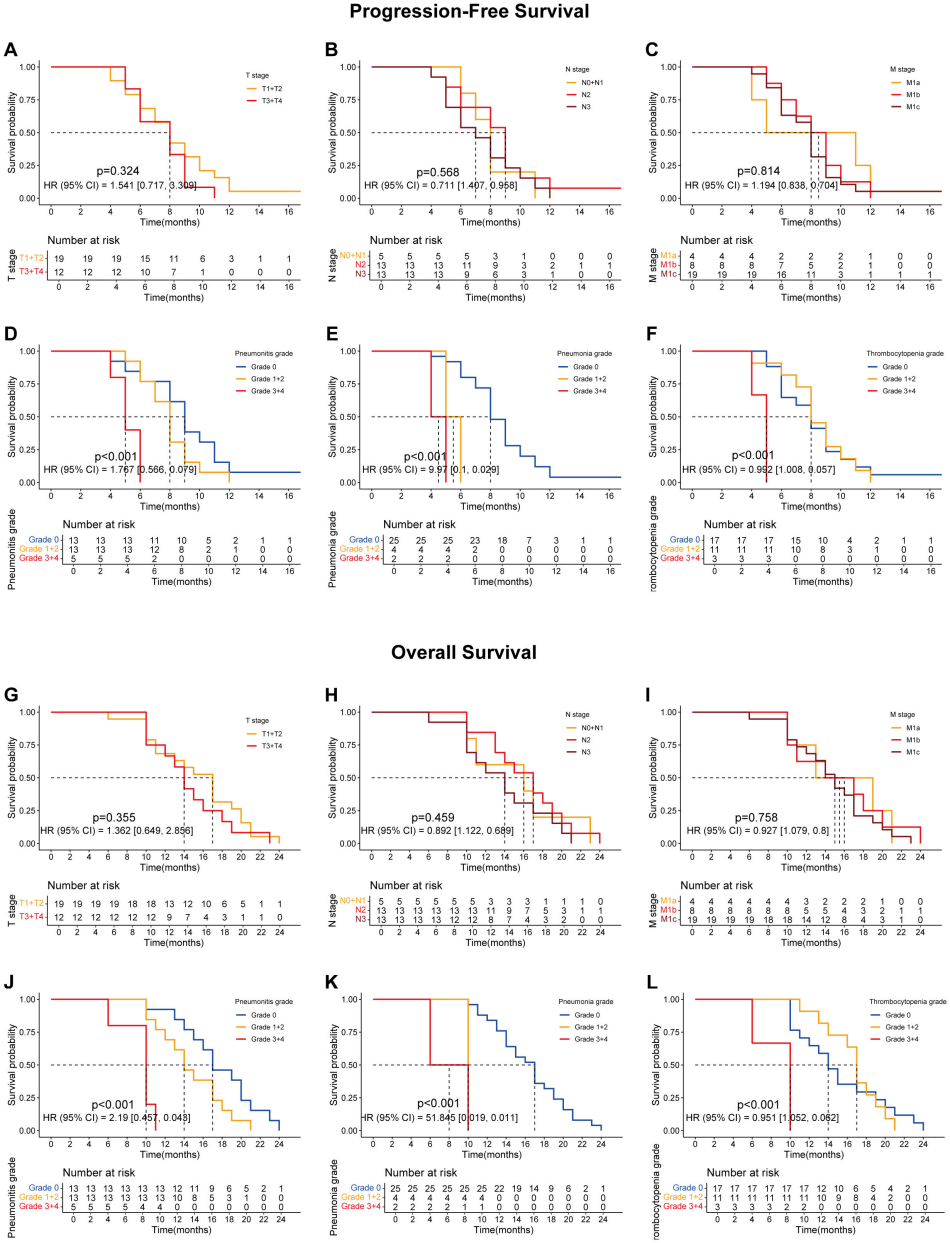
The survival curves showing the PFS and OS of lung cancer patients treated with the combination of ABCP but without radiotherapy. **(A)** PFS curves of patients with tumors at different T stages. The orange and red lines represent patients with stage T1–T2 and T3–T4 tumors, respectively. **(B)** PFS curves of patients with tumors at different N stages. The orange, red, and dark red lines represent patients with stage N0–N1, N2, and N3 tumors, respectively. **(C)** PFS curves of patients with tumors at different M stages. The orange, red, and dark red lines represent patients with stage M1a, M1b, and M1c tumors, respectively. **(D)** PFS curves of patients with different grades of pneumonitis. The blue, orange, and red lines represent patients with grade 0, grade 1–2, and grade 3–4 pneumonitis, respectively. **(E)** PFS curves of patients with different grades of pneumonia. The blue, orange, and red lines represent patients with grade 0, grade 1–2, and grade 3–4 pneumonia, respectively. **(F)** PFS curves of patients with different grades of thrombocytopenia. The blue, orange, and red lines represent patients with grade 0, grade 1–2, and grade 3–4 thrombocytopenia, respectively. **(G)** OS curves of patients with tumors at different T stages. The orange and red lines represent patients with stage T1–T2 and T3–T4 tumors, respectively. **(H)** OS curves of patients with tumors at different N stages. The orange, red, and dark red lines represent patients with stage N0–N1, N2, and N3 tumors, respectively. **(I)** OS curves of patients with tumors at different M stages. The orange, red, and dark red lines represent patients with stage M1a, M1b, and M1c tumors, respectively. **(J)** OS curves of patients with different grades of pneumonitis. The blue, orange, and red lines represent patients with grade 0, grade 1–2, and grade 3–4 pneumonitis, respectively. **(K)** OS curves of patients with different grades of pneumonia. The blue, orange, and red lines represent patients with grade 0, grade 1–2, and grade 3–4 pneumonia, respectively. **(L)** OS curves of patients with different grades of thrombocytopenia. The blue, orange, and red lines represent patients with grade 0, grade 1–2, and grade 3–4 thrombocytopenia, respectively. The x-axes indicate survival time, and the y-axes indicate survival probability. Grouping status is shown at the bottom of each chart. p < 0.05 in the log-rank test was considered statistically significant. PFS, progression-free survival; OS, overall survival; ABCP, atezolizumab, bevacizumab, carboplatin, and paclitaxel.

Preliminary analysis indicated that sequential radiotherapy, when added to the original quadruple therapy regimen, conferred a survival benefit (**Figure 1**; **Figures 1J–K**), particularly for overall survival (OS) (**Figure 1K**; *p* = 0.019). Whether in the experimental group, the control group, or the overall population, TNM staging remained the most significant factor affecting patients’ progression-free survival (PFS) and OS (**Figures 2**–**4**).

Further analyses revealed that certain treatment-related side effects, such as pneumonitis (**Figures 2D, J**, **3D, J**, **4D, J**), pneumonia (**Figures 2E, K**, **3E, K**, **4E, K**), and thrombocytopenia (**Figures 2F, L**, **3F, L**, **4F, L**), could affect patient prognosis—particularly when these side effects were greater than grade 3 (**Figures 2D-F, J-L**, **3D-F, J-L**, **4D-F, J-L**). Whether in the experimental group, the control group, or the overall population, TNM staging remained the most significant factor affecting patients’ progression free survival (PFS) and OS (**Figures 2A-C, G-I**, **Figures 3A-C, G-I**, **Figures 4A-C, G-I**).

**Figure 5:**
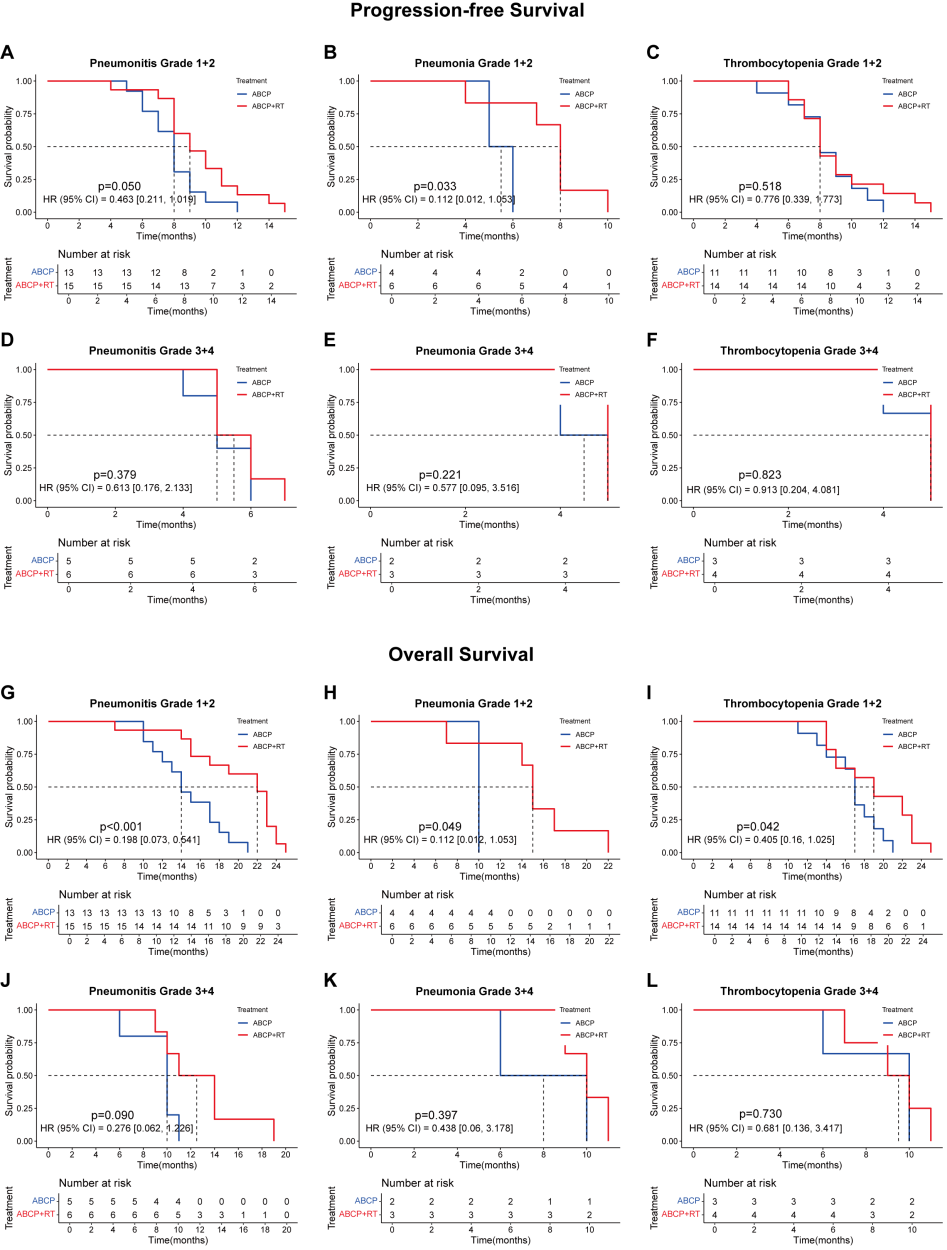
The association between the treatment, the PFS and the OS of patients with adverse reactions at different grades. **(A-C)** are the PFS curves of patients with grade 1 + 2 of pneumonitis **(A)**, pneumonia **(C)** and thrombocytopenia **(E)** after treated with ABCP or ABCP combined with RT. **(D-F)** are the PFS curves of patients with grade 3 + 4 of pneumonitis **(D)**, pneumonia **(E)** and thrombocytopenia **(F)** after treated with ABCP or ABCP combined with RT. **(G-I)** are the OS curves of patients with grade 1 + 2 of pneumonitis **(G)**, pneumonia **(H)** and thrombocytopenia **(I)** after treated with ABCP or ABCP combined with RT. **(J-L)** are the OS curves of patients with grade 3 + 4 of pneumonitis **(J)**, pneumonia **(K)** and thrombocytopenia **(L)** after treated with ABCP or ABCP combined with RT. The blue curves represent patients receiving only ABCP, while the red curves represent patients receiving ABCP combined with RT. The abscissa axes show survival time, while the ordinate axes show survival probability. The grouping status of the patients is indicated at the bottom of the chart. p< 0.05 in the Log-rank test was considered statistically significant. PFS, progression-free survival; OS, overall survival; RT, radiotherapy; ABCP, atezolizumab, bevacizumab, carboplatin, and paclitaxel.

Further analysis of treatment-related side effects showed that the lower the severity of side effects, the greater the survival benefit of radiotherapy (**Figure 5**). These trends were observed in both progression-free survival (PFS; **Figures 5A, B**) and overall survival (OS; **Figures 5G, H**).

## Discussion

For patients with EGFR mutation-negative NSCLC, the advent of immune checkpoint inhibitor (ICI) immunotherapy has revolutionized treatment and greatly improved prognosis (5). However, the overall survival (OS) of these patients remains short, indicating a need for improved treatment options. Notably, not all patients benefit from ICI therapy, and studies have shown that only 17%–21% of NSCLC patients respond to ICIs (21). Therefore, there is an unmet need to explore alternative combination strategies with tolerable toxicity to further improve response rates and prolong progression-free survival (PFS) and OS in patients with advanced NSCLC.

Reports of ICIs combined with chemoradiotherapy are common, but PD-L1 inhibitors are more frequently used in these regimens (7–9, 11). For patients with advanced NSCLC who have received a quadruple regimen primarily based on PD-1 inhibitors, whether adding radiotherapy confers survival benefit remains unclear. To date, few studies have addressed this. Radiotherapy (RT) has been shown to activate CD8^+^ T cells by inducing interferon-mediated CXCL10 and ICAM-1 expression in tumors, enhancing CD8^+^ T cell–tumor adhesion and recognition (22). However, there is still a lack of clinical research on combining radiotherapy, anti-angiogenic therapy, and PD-1 inhibitors in NSCLC treatment. This study was designed to explore the significance of incorporating radiotherapy into such combination therapies.

The original intention of this study was to improve the quality of life and prognosis of patients with slow tumor progression but stable disease (SD) status. Thoracic radiotherapy was administered after at least two cycles of quadruple therapy or when the patient’s efficacy evaluation showed SD with a trend of tumor enlargement. Even with a sequential combination approach, treatment-related side effects in the experimental group were significantly higher than in the control group (**Table 2**). Despite supportive use of hormonal drugs, expectorants, antiasthmatics, and blood-activating agents during radiotherapy, the risk of radiation-related pneumonitis remained significantly higher in the experimental group (p < 0.001), which was consistent with previous studies (8, 9, 11–14). These findings suggest that clinicians should pay special attention to the risk of pneumonitis and provide preventive supportive treatment as early as possible.

Although patients undergoing radiotherapy experienced more pronounced toxic side effects, their prognostic survival—especially OS—was significantly improved (**Figure 1**). A comprehensive subgroup analysis was conducted for treatment-related adverse reactions, and the survival prognosis results are summarized (**Figures 2**-**5**). While TNM staging remained the major factor affecting survival (**Figures 2**-**4**), certain side effects were identified as negative prognostic indicators (**Figures 2**-**5**), particularly when the grade of side effects increased. This trend was observed in both the overall population and subgroup analyses (**Figures 2**-**4**). In other words, patients with grade 1–2 side effects had a significantly better prognosis than those with grade 3–4 side effects (**Figures 2D-F, J-L**, **3D-F, J-L**, **4D-F, J-L**). When certain side effects reached grade 3–4, the survival benefit of radiotherapy became less pronounced (**Figures 5D–F, J–L**). This highlights the need for timely prevention and management of radiotherapy-related side effects (**Figure 5**) to help patients achieve better outcomes.

Pneumonitis can be caused by both radiotherapy and PD-1 inhibitors. In this study, the incidence was significantly higher than in previous studies on PD-L1 inhibitors combined with radiotherapy (8, 9, 11–14). It has been reported that reduced lung tolerance may result from PD-1–mediated PD-L2 blockade (23). Pneumonitis is a major factor limiting the total radiation dose (24). Therefore, pneumonitis should be actively prevented or managed during radiotherapy to avoid interruptions and to minimize its impact on patient prognosis and survival (**Figures 2D, J**, **3D, J**, **4D, J**, **5A, D, G, J**). Similarly, pneumonia and thrombocytopenia should also be taken seriously due to their negative prognostic effects (**Figures 2E, F, K, L**, **3E, F, K, L**, **4E, F, K, L**, **5B, C, E, F, H, I, K, L**).

Certainly, this study has limitations. First, it is a retrospective study, and inherent bias may be unavoidable. Prospective trials are needed to further validate these results. Second, the sample size is relatively small, which may affect the accuracy of findings. Additional patient data will be collected in future studies to strengthen the evidence. Third, the follow-up period is relatively short, and we will continue to monitor the clinical outcomes of surviving patients.

In summary, sequential radiotherapy based on a quadruple combination regimen can provide survival benefits to patients with advanced NSCLC while allowing for effective control of treatment-related adverse events.

## Conclusions

The findings suggest that sequential radiotherapy following quadruple therapy can prolong the survival of patients with advanced NSCLC. However, special attention should be paid to treatment-related side effects such as pneumonitis, pneumonia, and thrombocytopenia. These findings should be regarded as investigational until validated by larger prospective trials.

## Data Availability

The original contributions presented in the study are included in the article/**Supplementary Material**. Further inquiries can be directed to the corresponding author.

## Supplementary material

Supplementary Table 1 The clinical characteristics of patients involved in this study.

Supplementary Figure 1 PFS and OS of patients in different subgroups. **(A–C)** PFS curves of patients grouped by histological subtype **(A)**, PD-L1 expression level **(B)**, and smoking status **(C)**. **(D–F)** OS curves of patients grouped by histological subtype **(D)**, PD-L1 expression level **(E)**, and smoking status **(F)**. Grouping status is shown at the bottom of each chart. p < 0.05 in the log-rank test was considered statistically significant. PFS, progression-free survival; OS, overall survival.

## References

[1] Han B Zheng R Zeng H Wang S Sun K Chen R. Cancer incidence and mortality in China, 2022. J Natl Cancer Cent. (2024) 4:47–53. doi: 10.1016/j.jncc.2024.01.006, PMID: 39036382 PMC11256708

[2] Mok T Nakagawa K Park K Ohe Y Girard N Kim HR. Nivolumab plus chemotherapy in epidermal growth factor receptor-mutated metastatic non-small-cell lung cancer after disease progression on epidermal growth factor receptor tyrosine kinase inhibitors: final results of checkMate 722. J Clin Oncol. (2024) 42:1252–64. doi: 10.1200/JCO.23.01017, PMID: 38252907 PMC11095864

[3] Król K Mazur A Stachyra-Strawa P Grzybowska-Szatkowska L. Non-small cell lung cancer treatment with molecularly targeted therapy and concurrent radiotherapy-A review. Int J Mol Sci. (2023) 24:5858. doi: 10.3390/ijms24065858, PMID: 36982933 PMC10052930

[4] Garon EB Hellmann MD Rizvi NA Carcereny E Leighl NB Ahn MJ. Five-year overall survival for patients with advanced non–Small-cell lung cancer treated with pembrolizumab: results from the phase I KEYNOTE-001 study. J Clin Oncol. (2019) 37:2518–27. doi: 10.1200/JCO.19.00934, PMID: 31154919 PMC6768611

[5] Reck M Mok TSK Nishio M Jotte RM Cappuzzo F Orlandi F. Atezolizumab plus bevacizumab and chemotherapy in non-small-cell lung cancer (IMpower150): key subgroup analyses of patients with EGFR mutations or baseline liver metastases in a randomised, open-label phase 3 trial. Lancet Respir Med. (2019) 7:387–401. doi: 10.1016/S2213-2600(19)30084-0, PMID: 30922878

[6] Antonia SJ Villegas A Daniel D Vicente D Murakami S Hui R. Durvalumab after chemoradiotherapy in stage III non-small-cell lung cancer. N Engl J Med. (2017) 377:1919–29. doi: 10.1056/NEJMoa1709937, PMID: 28885881

[7] Daido W Masuda T Imano N Matsumoto N Hamai K Iwamoto Y. Pre-Existing Interstitial Lung Abnormalities Are Independent Risk Factors for Interstitial Lung Disease during Durvalumab Treatment after Chemoradiotherapy in Patients with Locally Advanced Non-Small-Cell Lung Cancer. Cancers (Basel). (2022) 14:6236. doi: 10.3390/cancers14246236, PMID: 36551721 PMC9776853

[8] Saito G Oya Y Taniguchi Y Kawachi H Daichi F Matsumoto H. Real-world survey of pneumonitis and its impact on durvalumab consolidation therapy in patients with non-small cell lung cancer who received chemoradiotherapy after durvalumab approval (HOPE-005/CRIMSON). Lung Cancer. (2021) 161:86–93. doi: 10.1016/j.lungcan.2021.08.019, PMID: 34543942

[9] Shintani T Kishi N Matsuo Y Ogura M Mitsuyoshi T Araki N. Incidence and risk factors of symptomatic radiation pneumonitis in non-small-cell lung cancer patients treated with concurrent chemoradiotherapy and consolidation durvalumab. Clin Lung Cancer. (2021) 22:401–10. doi: 10.1016/j.cllc.2021.01.017, PMID: 33678582

[10] Zhang T Xu K Bi N Zhang L Jiang W Liang J. Efficacy and safety of immune checkpoint inhibitor consolidation after chemoradiation in patients of Asian ethnicity with unresectable stage III non-small cell lung cancer: Chinese multicenter report and literature review. Thorac Cancer. (2020) 11:2916–23. doi: 10.1111/1759-7714.13631, PMID: 32833338 PMC7529561

[11] Dang J Li G Zang S Zhang S Yao L. Risk and predictors for early radiation pneumonitis in patients with stage III non-small cell lung cancer treated with concurrent or sequential chemoradiotherapy. Radiat Oncol. (2014) 9:172. doi: 10.1186/1748-717X-9-172, PMID: 25074618 PMC4120001

[12] Li F Zhou Z Wu A Cai Y Wu H Chen M. Preexisting radiological interstitial lung abnormalities are a risk factor for severe radiation pneumonitis in patients with small-cell lung cancer after thoracic radiation therapy. Radiat Oncol. (2018) 13:82. doi: 10.1186/s13014-018-1030-1, PMID: 29716649 PMC5930768

[13] Vogelius IR Bentzen SM. A literature-based meta-analysis of clinical risk factors for development of radiation induced pneumonitis. Acta Oncol. (2012) 51:975–83. doi: 10.3109/0284186X.2012.718093, PMID: 22950387 PMC3557496

[14] Lu C Lei Z Wu H Lu H. Evaluating risk factors of radiation pneumonitis after stereotactic body radiation therapy in lung tumor: Meta-analysis of 9 observational studies. PloS One. (2018) 13:e0208637. doi: 10.1371/journal.pone.0208637, PMID: 30521600 PMC6283643

[15] Hodi FS Ballinger M Lyons B Soria JC Nishino M Tabernero J. Immune-modified response evaluation criteria in solid tumors (imRECIST): refining guidelines to assess the clinical benefit of cancer immunotherapy. J Clin Oncol. (2018) 36:850–8. doi: 10.1200/JCO.2017.75.1644, PMID: 29341833

[16] Hagelstein V Ortland I Wilmer A Mitchell SA Jaehde U. Validation of the German patient-reported outcomes version of the common terminology criteria for adverse events (PRO-CTCAE™). Ann Oncol. (2016) 27:2294–9. doi: 10.1093/annonc/mdw422, PMID: 27681863 PMC6267864

[17] Basch E Dueck AC Rogak LJ Mitchell SA Minasian LM Denicoff AM. Feasibility of implementing the patient-reported outcomes version of the common terminology criteria for adverse events in a multicenter trial: NCCTG N1048. J Clin Oncol. (2018) 36:JCO2018788620. doi: 10.1200/JCO.2018.78.8620, PMID: 30204536 PMC6209091

[18] Torjesen I. Combination immunotherapy: the emerging treatment that removes cancer’s “cloak of invisibility. BMJ. (2019) 365:l1824. doi: 10.1136/bmj.l1824, PMID: 31040106

[19] Venkatesan P. Durvalumab lengthens survival in patients with NSCLC. Lancet Respir Med. (2017) 5:850. doi: 10.1016/S2213-2600(17)30353-3, PMID: 28943343

[20] American Association for Cancer Research. Durvalumab extends OS in NSCLC. Cancer Discov. (2018) 8:OF5. doi: 10.1158/2159-8290.CD-NB2018-135, PMID: 30327351

[21] Aslan V Yazici O Zdemir N. Increased tumor mutation burden levels and sensitivity of non-small cell lung cancer to PD-L1 blockade. JAMA Oncol. (2023) 9(4):570. doi: 10.1001/jamaoncol.2022.7586, PMID: 36729466

[22] Wang CL Ho AS Chang C Sie ZL Peng CL Chang J. Radiotherapy enhances CXCR3highCD8+T cell activation through inducing IFNγ-mediated CXCL10 and ICAM-1 expression in lung cancer cells. Cancer Immunol Immunother. (2023) 72:1865–80. doi: 10.1007/s00262-023-03379-6, PMID: 36688994 PMC10198930

[23] Ando H Suzuki K Yanagihara T. Insights into potential pathogenesis and treatment options for immune-checkpoint inhibitor-related pneumonitis. Biomedicines. (2021) 9:1484. doi: 10.3390/biomedicines9101484s, PMID: 34680601 PMC8533467

[24] Jain V Berman AT. Radiation pneumonitis: old problem, new tricks. Cancers (Basel). (2018) 10:222. doi: 10.3390/cancers10070222, PMID: 29970850 PMC6071030

