# Supplementary figures and images for: Efficacy and safety of thoracic radiotherapy combined with anti-angiogenic therapy and immunochemotherapy for advanced non-small cell lung cancer patients: a retrospective study
Source: Front Oncol. 2025 Sep 23;15:1640306. doi: 10.3389/fonc.2025.1640306 (PMC12500576; doi:10.3389/fonc.2025.1640306)

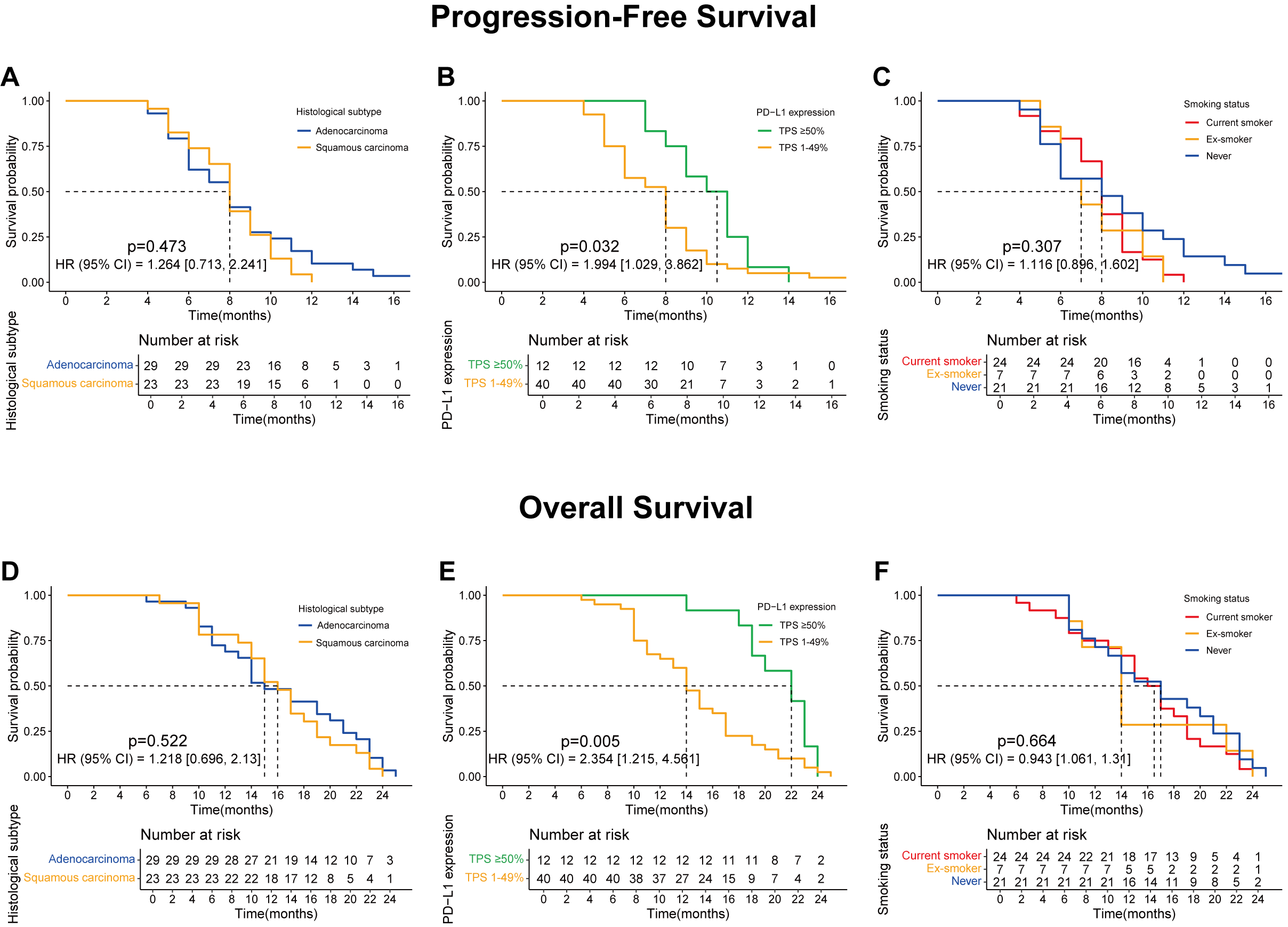

Supplement: Supplementary Figure 1 — PFS and OS of patients in different subgroups. (A–C) PFS curves of patients grouped by histological subtype (A), PD-L1 expression level (B), and smoking status (C). (D–F) OS curves of patients grouped by histological subtype (D), PD-L1 expression level (E), and smoking status (F). Grouping status is shown at the bottom of each chart. p < 0.05 in the log-rank test was considered statistically significant. PFS, progression-free survival; OS, overall survival. [file Image1.tif]
